# Dr. Patterson, on the Use of Digitalis

**Published:** 1801-05

**Authors:** W. Patterson

**Affiliations:** Londonderry


### *To the Editors of the Medical and Physical Journal.*

GENTLEMEN,

A Work purſued with zeal, conducted with candour, and executed with ability, at once intereſts the reader, and claims his beſt endeavours toward its ſupport. Give me leave, in uniſon with multitudes of my brethren, to attach this character to your Journal; and in offering this equitable tribute to its merits, allow me to preſent to you for publication the following caſes, as ſamples whereby to judge in what degree this tribute ſhould be appreciated. I muſt firſt, however, felicitate the ingenious and liberal-minded of the Faculty on their having a mode of conveying their ſentiments to the world ſo favourable and ready as your Journal manifeſtly affords; ſeeing that they muſt be ſenſible, that it is a duty which they owe to ſociety (in return for what they reap) to make public, without reſerve and without delay, the harveſt of their labours, whether theoretical or practical, provided it tend to throw additional light on the nature and cure of diſeaſes.

The caſes treating of *Digitalis* are ſelected, on account of medical medical practitioners being at present remarkably occupied about the operative and curative properties of this plant. The last case is communicated as being curious and extraordinary, not having, within the compass of my reading and recollection, a parallel in the records of Medicine. I am, &c.

*Londonderry, March* 31, 1801. W. PATTERSON.

---

CASE I.

ISABELLA M'GOWAN, aged 24 years, admitted into the Hospital January 4, 1796, since the preceding September has been gradually growing melancholy and sullen; is choleric and intractable with her kindred, but pacific and manageable amongst strangers; her countenance is gloomy and peculiarly glouting; pulse regular; bowels natural; and, if she do not sleep, at least she lies quiet in bed at night. She had lain-in about two months before the approaches of mental derangement were noticed; soon afterwards her husband emigrated to America, leaving her burthened with three young children; the breast-milk suddenly vanished; and she had disputes with the husband's relations respecting some property he left behind. She is not emaciated; says she has no ailment; but her answers are generally very indistinct and incongruous.

A variety of medicines, as purges, vomits, blisters, opiates, fetids, and mercurials to the extent of salivation, were employed with very little advantage from the time of admission until the 11th of May following, when the use of *Digitalis* was commenced, and two grains administered, morning and night, till eight doses were taken, without producing either visible effect or operation. The dose was gradually augmented, until it was brought to eight grains twice a day; but being withdrawn from the hospital at this juncture, no satisfactory observation could be made on the operation of the medicine, which, however, was continued at home, where, in a short time, she showed signs of returning reason, which was restored by degrees, and which happy change her friends ascribed to perseverance in the employment of the Digitalis. This patient did not require a strait-waistcoat.

CASE II.

MARGARET HENEY, aged 37 years, admitted into the hospital May 28, 1799, was delivered of a child six months past, and four days after delivery got a fright, which was succeeded by a tedious fever. About three weeks ago she was attacked with with infanity. Her pulfe is fmall and flow; countenance pale and dejected; not furious, but very talkative; has fome periods of rationality; fpeaks on certain topics with tolerable fenfe; and her memory is not much enfeebled.

As in the cafe of M'Gowan, fundry medicines were employed without material benefit during the fpace of a month, at the end of which *Digitalis* was prefcribed, and near three grains were given morning and evening for thirty-nine days; in the courfe of which, two grains of opium, combined with calomel and antimonial tartar, were given at bed-time, to obviate fleeplefs nights. Obferving no curative effect from thefe meafures, a large plafter of cantharides was applied to the head, and four grains of opium were ordered at night; and as this alfo availed little, a drachm of etherial vitriolic fpirit, mixed with forty gts. of tinct. opii, was directed to be given three times a day; and afterwards the fcalp was covered with another bliftering plafter.

The patient was now taken home, and her friends were enjoined to give her at night, two grains of opium, in conjunction with three of afafœtida, and one and a half of calomel. In feventeen days a ptyalifm was produced; fhe fometimes attended to domeftic bufinefs, but in general was inattentive, and fometimes outrageous. When the falivation abated, *ten* grains of Digitalis with fix of ginger were ordered to be given three times a day. The 2d of October her hufband came to the hofpital to report the effects of the prefcription, which, he faid, were really extraordinary. One or two dofes operated violently both by purging and vomiting, caufed dimnefs of fight and pain in the head, attended with fuch extreme debility that fhe thought herfelf dying, and at the fame time declared that fhe felt her fenfes (as fhe expreffed it) perfectly reftored, which her hufband affirmed was actually the cafe, whilft under the influence of the medicine. But no fooner did this influence decay than the derangement returned, accompanied with the fame loquacious propenfity, and often with paroxyfms of rage.

Although the primary operation of the medicine was fo defperate, its ufe was cautioufly continued until the number of dofes, eighteen, which had been prefcribed, were confumed, but without producing equally lucid intervals. No farther report was received till the 13th of November, when circumftances were recounted refembling the preceding in moft refpects, except that the gleams of intellect were not fo obfervable as on the firft adminiftration. Nine dofes of the herb were then given; and, having had no fucceeding intelligence of the patient, I conclude that her reafon was retrieved, otherwiſe her friends, who were very ſolicitous to give her medicine, would not have ceaſed coming for ſupplies. This lunatic frequently required confinement in a dark cell, and ſometimes the coercion of a ſtrait-waiſtcoat.

### CASE III.

The effects of Digitalis in the two preceding caſes, encouraged me to try it in the caſe of Cath. M'Dade, a married woman, aged about 30 years, whoſe derangement had ſubſiſted two or three years before her introduction into the hoſpital. When ſhe was brought to the houſe, a very imperfect account was given of her previous ſituation; but it then appeared, that her inſanity was very different from the melancholy ſpecies; for ſhe was garrulous in the extreme, prone to inceſſant dancing and ſinging, with a briſk penetrating eye, and a bold deportment, but not a miſchievous diſpoſition; always happy, and never complaining. Her appetite was good, and her ſleep generally regular. The only exciting cauſe of her malady that I could diſcover, came out when her reaſon was in ſome meaſure reclaimed, which was, as herſelf related, an imputation againſt her fidelity to the marriage vow.

Nearly three grains of Digitalis were adminiſtered thrice a day, for the ſpace of ſix days, at the expiration of which, tokens of returning reaſon appeared, but the operative effect of the plant could not be well aſcertained. However, the remedy was perſiſted in for ſome weeks, and the conſequence was a revival of rationality in as great a degree as her conſtitution will admit, by which ſhe is rendered capable of ſpinning, waſhing, and doing other ſervile offices in the houſe.

### CASE IV.

In March, 1800, H. T. Eſq. aged 62 years, middle ſtature, wan viſage, ſpare ſhape, diligent, and much expoſed to cold and fatigue, had a ſharp pneumonic inflammation, from which he was reſcued by one moderate veneſection, a bliſter, calomel, purges, acetated ammonia, ſmall doſes of tartariſed antimony, gum arabic, diluting drinks, and vegetable aliment. Shortly after recovery from this diſorder, his feet began to ſwell when ſitting out of bed; the ſwelling ſoon extended half way up his legs, and in two or three days more, it proceeded to the trunk. On examination, the ſwelling in the lower extremities was found to be anaſarcous, and that in the body ſeemed to be of a ſimilar nature, as no fluctuation could be diſtinguiſhed in the cavity of the abdomen. He had not any thirſt; no paucity of urine, though what he paſſed was high coloured; coloured; ſlept well; ſcarcely any cough; no pain in either ſide of the thorax; and his appetite, eſpecially for dinner, was pretty good.

The uſe of cryſtals of tartar, and the aſſiduous application of frictions, were then recommended, and purſued ſome days; but not being attended with ſufficient advantage, tincture of jalap was added to the cryſtals, ſo as to operate briſkly on the inteſtines, interpoſing between the purges, calomel, nitrat of potaſh, ſquills, ſp. ætheris nitroſi, bitters, and infuſions of horſe-radiſh, juniper berries, &c. By theſe means, the tumefactions were reduced in the courſe of two months, and he continued in tolerable health until the latter end of Auguſt, when ſymptoms of the ſame diſeaſe appeared anew, advancing in a pace not to be reſtrained.

On this occaſion of relapſe, not only were employed the generality of the former remedies, but alſo ſome additional ones, amongſt which were different forms of *Digitalis Purpurea*. The firſt preparation of it was the tincture, which was commenced the 9th of November, and which was ordered to be taken, at firſt, in the quantity of fifteen drops twice a day, increaſing five drops each doſe, till they amounted to thirty-five drops; and this number was at length given thrice a day for a few days. After the operation of a purge, an infuſion of the Digitalis was entered on, taking an ounce every ſixth or eighth hour. No advantage being gained by this preparation of the herb, another purge was taken, and a decoction of the freſh leaves was preſcribed; a large table ſpoon full every ſecond hour, for four, ſix, or more times, provided neither ſtomach ſickneſs, dizzineſs, vomiting, diureſis, nor purging would happen to curtail its exhibition. Though a ſenſible impreſſion was the conſequence of the firſt or ſecond doſe, he perſevered until four were taken, when he experienced vaſtly diſtreſſing affections, ſuch as heavy ſtomach ſickneſs, frequent vomiting, confuſion in the head, dimneſs of ſight, and ſtops in the action of the heart. The pulſe of courſe was very unequal and intermittent; there was a great drowſineſs; conſiderable debility; and, what I do not recollect to have before witneſſed, or heard of, as an effect of Digitalis, a moſt irkſome pain in the gums and teeth during ſome hours. The narcotic influence was ſevere for two days, after which it gradually retired, and the preceding general medicines were reſumed, with the addition of mercurial frictions, but with no better reſult.

At length, the tenſion and bulk of the abdomen becoming inſupportable, attended with ſymptoms of increaſing debility, the paracenteſis was performed the 29th of December; thirtyone one pints of fluid were extracted, and he underwent the evacuation without faintnefs in fpirits or ftrength. A few hours after the operation, a languor took place, and he continued in a flat, exhaufted alarming ftate during twelve days, when he gained a little ground, and feemed likely to furvive fome time. At this juncture, recourfe was had to preparations of fteel, mineral alkali, muriatic acid, mild laxatives, &c. but the morbid accumulation in the abdomen advanced, notwithftanding every endeavour to oppofe it. His hopes were now greatly raifed by extravagant encomiums that were lavifhed on a ruftic noftrum, the trial of which he therefore commenced, and perfifted in the ufe of it fo long, that the harbingers of fpeedy diffolution precluded the repetition of the palliative of tapping, and the mortal cataftrophe happened the 7th of March, 1801.

### CASE V.

Mrs. C——, aged about 40 years, a widow, of pale complexion, auburn hair, dark brown eyes, became in the winter, 1799, fenfible of the beginning of an illnefs, which fhe confidered a common cold, and for which fhe had recourfe to the advice of an apothecary, who treated her with the drugs commonly denominated pectorals. Under this adminiftration fhe continued eight or ten weeks, when I was required to vifit the unrelieved patient, and found her affected with quick and uneafy refpiration, frequent dry cough, frothy tenacious expectoration, quick wiry pulfe, impaired whimfical appetite, fits of vomiting, thirft, and copious morning fweats: but in no part of the thorax did fhe experience or confefs to feel any pain or ftricture. My prefcriptions were principally one moderate venefection, two fmall blifters, minute dofes of tartarifed antimony, fulphuric acid, gum arabic, honey, oil, and as much animal nourifhment as the palate and ftomach would admit.

Proceeding feveral weeks on the principles which dictated thofe meafures, and feeing that no ground was gained, except by the difeafe, which rapidly advanced, I determined to try the virtues of Digitalis. Accordingly, as the ftomach of this patient was delicate and irritable, I began, early in April, 1800, with twelve drops of the tincture twice a day, and gradually increafed the quantity until it arofe to eighteen drops, which produced fuch diforder in her ftomach, that fhe could not again be prevailed upon to take more than ten or twelve drops for a dofe; and foon refufing to make trial even of this number, fhe ultimately relinquifhed this, and every other medicine, together with all medical advice. Indeed, there are juft grounds for believing that this was a loft cafe before before I was called in, as the difeafe then affumed a formidable afpect, and yielded not a ftep to any effort made againft its progrefs, but went on inflexibly until it configned the fufferer, the middle of the following month, to the cold embraces of the king of terrors.

### CASE VI.

June the 2d, 1800, Mary Smyth, aged 60 years, began to take Tinct. Digitalis, which fhe was directed to do thrice daily, thirty drops for each dofe, in plain water. At this time fhe laboured under a defperate fit of afthma. Her breathing was extremely laborious, feemingly fuffocating; her face bluifh and bloated; the ends of her fingers were livid; pulfe in the wrift fcarcely perceptible, whilft the carotids throbbed moft violently; hardly able to cough; expectoration nearly fufpended; trunk of the body fwelled; and the lower extremities very anafarcous. The firft dofe of the Digitalis affuaged the throbbings of the carotids, the fecond produced fome diuretic effect, and the third caufed a confiderable flow of urine, fo copious and fo conftant, that the patient was alarmed at the evacuation, whilft fhe was aftonifhed at the complete and fudden reduction of the difeafe, done, as it were, by a charm. The medicine, at this juncture, produced fo little ftomach ficknefs, that fhe was able to purfue the ufe of it, without interruption, until about an ounce was confumed, at which time fhe was evidently convalefcent, and fhortly afterward, by means of diet, and a cautious ufe of the open air, her health was as fully reftored as her conftitution reafonably would admit.

Since that falutary change, fhe has had fome threatenings of relapfe, but the progrefs of thefe always have been checked by refuming the Digitalis, which, on thefe latter occafions, has induced more naufea and ftomach affection than it did at firft; confequently, at each of thofe latter adminiftrations, the ufe of it was fooner fufpended. In October, fhe was fo well and ftrong as to be able to rife in the night, and act as a nurfe-tender to a lying-in friend, without receiving any injury to her health from the expofure. In December, fome threatenings of the dyfpnœa induced her to refort again to the Digitalis, the occafional ufe of which keeps the diforder at bay. In February, 1801, fhe had an attack of the catarrhus fenilis, from which fhe recovered fo well as to be able at prefent to move about the houfe.

### CASE VII.

Lieut. Col. R——, aged 28 years, middle ftature, flim fhape, fallow complexion, and liable to retrograde actions of the alimentary mentary canal. I was called to vifit, June 13, 1800, and found him in bed, affected as follows: A confiderable tumefaction and tenfion in the abdomen, principally in the epigaftric region, attended with great fenfibility on the moft gentle preffure, whilft the region of the bladder, as he lay in bed, was in no degree diftended nor painful to the touch; languor, faintifh ficknefs, when in an erect pofture; and confiderable thirft. The pulfe quick and unequal, generally fmall, but fometimes a few ftrokes pretty round and full. Frequent defire to difcharge the contents of the bladder, but without any perceptible evacuation of urine, coagulated blood being the only apparent excretion, which was fometimes voided in remarkable quantity when fitting on a night-chair. The preceding day he had been expofed to wet clothes, whilft heated by field-exercife, after which he drank copioufly of cyder and wine, (to which he was addicted) but without any of the mefs, in this inftance, having heard him complain of a retention of urine. Thus inebriated, he fallied forth to collect the regimental band, for the purpofe of ferenading; and, it is affirmed, that he attempted hoifting on his fhoulders one of his companions, to tap at the window of another officer, in order to get him to make one of the party. In the moment of this exertion, or foon afterward, it was reprefented that he grew extremely fick, from which he fhortly was relieved by vomiting. However, he was conducted to his lodgings in a very weak, oppreffed condition, accompanied with a moft profufe fweat, which continued fome hours.

Mr. Purdon, ftaff-furgeon of the diftrict, an experienced practitioner, was now fent for, and he drew a little blood from the arm, ordered an enema, and prefcribed a dofe of Oleum Ricini. Three or four hours afterward, I faw the patient, and finding that the oil was vomited, and the enema had no operation, whilft the unfpeakable diftrefs attending the above fymptoms continued, I advifed fomentations to the abdomen, and another venefection; in conjunction with which, were directed ftrong purgative pills, compofed of ext. colocynth. comp. and calomel. Thefe meafures produced no good effect; and accordingly, enemas were repeatedly adminiftered next day, together with a fcruple of calomel, given by the mouth in two dofes. A warm bath, by means of the flipper machine, was, on the third morning, employed, and borne very well, yet unattended with any fervice; a blifter was applied on the epigaftrium; more cathartic pills were prefcribed; and ftrong mercurial ointment was diligently rubbed in on the trunk and extremities. Thefe were followed by a variety of injections, oleagenous, fœtid, and ftimulating, without procuring any evacuation of fæces; at length, upon giving one or two aloetic pills, a difcharge of fluid fluid excrement commenced, and continued in the form of diarrhœa for feveral hours. Mucilaginous, oily, and ætherial preparations, with mild opiates, were occafionally taken into aid; but, notwithftanding the moft fedulous attention, and the moft active treatment, he paid the laft debt of nature in this fublunary ftate, in the afternoon of the 16th, in an eafy, tranquil manner, as if life were abftracted by a profufe hæmorrhage.

The ambiguity in which this curious and intricate cafe was involved, led to a diffection, which was performed next day, of that part only, the abdomen, wherein the difeafe appeared to be feated. On opening the peritoneum, there iffued forth a confiderable quantity of a fluid refembling bloody ferum, the greater part of which being preferved, it was meafured, and was found to amount to five quarts. The inteftina tenuia, throughout their whole extent, exhibited a vafcular rednefs on the exterior tunic, in fome places to a confiderable degree, which plainly reprefented a ftate of inflammation, being manifeftly different from any dye that might be imparted by the extravafated fluid. The cæcum and head of the colon were highly inflamed, diftended, and fpotted with lividity; the reft of the colon and appendicula vermiformis were healthy. The ftomach, the liver with its appendages, and all the other abdominal vifcera, were perfectly found, except the *vefica urinaria*; on one fide of the fundus of which was difcovered an opening, about two-tenths of an inch, in each direction, with fome extenuation of the coats in this fpot, and confpicuous marks of inflammation furrounding the rupture. This breach in the bladder, at once decides the nature of the fluid that efcaped on the incifion of the peritonæum, namely, that it was urine tinged with blood.

On this occafion, fome queries occur, which I fhall put to your correfpondents, with a view to gain additional information, fhould ever fuch a formidable cafe come in my way hereafter. Was the malady curable? Or was it 'paft all furgery?' Was it attended with any circumftance conducting to a fatisfactory diagnofis? With refpect to this laft query, the reader may be reminded, as furnifhing a diagnoftic fymptom, that the patient being always examined in a recumbent pofture, no prominency was difcerned in the hypogaftric region, fuch as indicates a bladder diftended with urine from ifchury. Had examination fortunately been made in an erect fituation, would not a fagacious obferver, combining this with the other particulars in the preceding recital, be led to form an adequate idea of the real caufe of the calamity?

### *Hydrocephalus Internus.*

In the Third Vol. of the Medical Journal, p. 113, commences a refpectable communication, in which the author, Mr. White, of Bath, laments not only the frequent infidious and equivocal nature of the fymptoms of this difeafe, but alfo the oppofite opinions refpecting its proximate caufe, which are ftill entertained by medical writers. On this occafion, I take leave to refer Mr. White to a fmall Tract, which I publifhed at Gilbert's Medical Library, in Dublin, in the fummer of 1794, and in which he will find the characteriftic marks of the difeafe pointed out, the proximate caufe traced to inflammatory action, and other circumftances relating to it, afcertained in fuch manner as might have unravelled and obviated the perplexities which he deplores, had the Tract fallen in his way. There he would have feen to whom we are indebted for the germ of knowledge concerning the nature of the malady; for the firft hints of applying mercury in dropfical cafes as an agent on the abforbent powers; for the early ufe of that mineral as a remedy in the febrile difeafes of children, attended with a *lethargic dulness* and *sleepiness*; and for its ftill more early employment, in confiderable dofes, as a purgative. There he will fee, that the ideas affumed by Darwin, Beddoes, Rufh, &c. were conceived, explained, and laid before the public, prior to the publication of the fentiments of thofe diftinguifhed phyficians. Within the laft fix years, feveral more cafes and illuftrations have occurred to the writer of this article, which he hopes to have an opportunity of publifhing on fome future day.

